# Understanding the associations between neurodevelopmental features and internalising and externalising behaviours: A transdiagnostic approach

**DOI:** 10.1002/jcv2.70110

**Published:** 2026-03-08

**Authors:** Sarah J. Carrington, Jennifer Keating, Mirko Uljarević, Kirsten Abbot‐Smith, Catherine R. G. Jones, Susan R. Leekam

**Affiliations:** ^1^ School of Psychology Aston University Birmingham UK; ^2^ School of Social Sciences Cardiff University Cardiff UK; ^3^ Department of Psychiatry and Behavioral Sciences Stanford University Stanford California USA; ^4^ School of Psychology University of Kent Canterbury UK; ^5^ Cardiff University Centre for Human Developmental Science School of Psychology Cardiff University Cardiff UK

**Keywords:** externalising, internalising, pragmatic language, restricted and repetitive behaviours, social communication, transdiagnostic research

## Abstract

**Background:**

Internalising and externalising behaviours—significant markers for lifetime psychiatric vulnerability—are elevated in children with neurodevelopmental diagnoses, including autism. Although neurodevelopmental features of autism are dimensions that span the population, limited research has examined their differential patterns of association with internalising and externalising behaviours in children without specific, categorically‐defined diagnoses. Evidence of such associations outside of a traditional diagnostic context may enable more targeted support for children's individual needs, irrespective of diagnoses. The current study aimed to characterise the relationship between neurodevelopmental features found in autism—restricted and repetitive behaviours (RRB) and social communication difficulties—and internalising and externalising behaviours in children from mainstream school who experience emotional, behavioural, or cognitive challenges.

**Methods:**

We recruited 136 6–7‐year‐olds without known clinical conditions but with school‐identified emotional, behavioural or cognitive difficulties. The Repetitive Behaviour Questionnaire‐2 assessed RRBs, the pragmatics scale from the Revised Children's Communication Checklist‐2 assessed social communication, and the Strengths and Difficulties Questionnaire examined internalising and externalising behaviours.

**Results:**

Simultaneous hierarchical linear regression analysis identified differential associations when adjusting for covariance between internalising and externalising. Social communication made a stronger contribution than RRBs to variance in externalising behaviours (*F*change_(1, 131)_ = 11.84, *p* < 0.001). However, for internalising behaviours, RRBs made the strongest contribution (*F*change_(2, 131)_ = 8.19, *p* < 0.001). The insistence on sameness subdomain of RRB predicted variance in internalising but not externalising behaviours independently of social communication while the repetitive sensory and motor behaviour subdomain predicted variance in externalising but not internalising behaviours, but only when social communication was not included.

**Conclusion:**

These findings will inform future research aimed at understanding the co‐occurrence of traits across diagnostic boundaries. Evidence that RRBs and social communication are differentially associated with internalising and externalising behaviours may identify target areas for the support of children with emotional and behavioural difficulties, a group whose co‐occurring neurodevelopmental features are often under‐recognised.

## Key Points

**What's Known?**

- Internalising and externalising behaviours are elevated in autism, but no research has examined their differential, simultaneous associations with neurodevelopmental features of autism in other children.

**What's New?**

- This study adopted a transdiagnostic sampling approach to examine differential associations between neurodevelopmental features of autism (restricted and repetitive behaviours [RRBs] and social communication) and internalising and externalising behaviours.
- Social communication difficulties made the strongest contribution to externalising behaviours.
- RRBs—specifically insistence on sameness behaviours—made the strongest contribution to internalising behaviours.

**What's Relevant?**

- Our findings highlight the benefits of a dimensional, transdiagnostic research approach to inform educational and clinical practice, emphasising consideration of neurodevelopmental features in relation to emotional and behavioural difficulties rather than a diagnosis‐led approach to supporting children.

## INTRODUCTION

Internalising and externalising behaviours are significant markers for a child's lifetime vulnerability. Indeed, behaviours that form part of the internalising spectrum (e.g., fearfulness, social withdrawal) and externalising spectrum (e.g., conduct problems, hyperactivity) signal increased risk for psychiatric problems, poor wellbeing and quality of life, reduced academic success, and unemployment across the lifespan (Adriaanse et al., 2015; Aunola et al., 2000; Caspi et al., 1996; Vergunst et al., 2023). Research using traditional diagnostic categories (DSM‐5; American Psychiatric Association, 2013), shows that internalising and externalising behaviours are highly elevated in child samples selected with diagnosed neurodevelopmental conditions such as autism (Bauminger et al., 2010; Kim et al., 2000; Russell et al., 2013; Tsai et al., 2020) and attention deficit hyperactivity disorder (ADHD; Mlodnicka et al., 2025). However, the purpose of the current study was to understand the nature of the associations between neurodevelopmental features and internalising and externalising outside of a traditional diagnostic context.

Better understanding of the way in which neurodevelopmental features are associated with internalising and externalising behaviours has implications for dimensional research that is directed by transdiagnostic theoretical approaches, such as the Research Domains Criteria (RDoC; Cuthbert & Insel, 2013) and the Hierarchical Taxonomy of Psychopathology (HiTOP; Kotov et al., 2017), and that model psychiatric traits across diagnostic boundaries (Astle et al., 2022; Cuthbert & Insel, 2013; Kotov et al., 2017; Michelini et al., 2024). Improved understanding of the association between neurodevelopmental features and internalising and externalising behaviours also has practical implications for children's clinical and education services. More specifically, it offers an opportunity for better supporting children whose main presenting challenges are emotional and behavioural and who have co‐occurring neurodevelopmental features that are under‐recognised and under‐supported (Astle et al., 2022).

Thus, the current study adopted a dimensional transdiagnostic approach. While the term ‘transdiagnostic’ may imply comparison across different, diagnosed conditions, we use it here to follow one of several research methods outlined by Astle et al. (2022). Specifically, children in our study were selected not on the basis of any known diagnosis but on their functional need for assessment due to behavioural, emotional, and/or cognitive difficulties identified at school. The goal was to examine the inter‐relationship between internalising and externalising behaviours and specific neurodevelopmental features found in autism—restricted and repetitive behaviours (RRBs) and social communication. Although RRBs and social communication features are diagnostic criteria for autism (DSM‐5; American Psychiatric Association, 2013), both are dimensional constructs that form a continuum across the general population (Geurts & Embrechts, 2008; Leekam et al., 2007, 2011). Furthermore, while early twin studies indicated that these two domains are independent of each other, or are ‘fractionable’ (Happé et al., 2006), more recent research with both clinical and general population samples has shown that social communication and RRBs are strongly and specifically correlated with each other even in children without a diagnosis of autism (e.g., Frazier et al., 2014; Keating et al., 2024). Nevertheless, very few research studies have focused on the differential association between RRBs and social communication and internalising and externalising behaviours outside of categorically diagnosed groups. Therefore, the purpose of the current study was to focus for the first time on a non‐clinical, school‐identified sample to address both RRB and social communication domains simultaneously. Using a unique sample of 6‐ and 7‐year‐old children with school‐identified behavioural, emotional, and/or cognitive difficulties, we assessed specificity in these neurodevelopmental features in relation to internalising and externalising behaviours, while adjusting for the co‐occurring internalising and externalising behavioural dimensions.

The domain of RRBs incorporates multidimensional features, spanning repetitive motor behaviours (e.g., rocking, spinning), differences in sensory responsiveness, and fixation on routines and focused interests. A recent conceptual and quantitative overview of the RRB literature has identified two subdomains—repetitive sensory and motor behaviours (RSMB) and insistence on sameness (IS)—that most commonly emerge from factor analytical studies across both clinical and non‐clinical populations (Uljarević et al., 2023). While the association between IS and internalising is well established in autistic (e.g., Lidstone et al., 2014; Rodgers et al., 2012) and non‐autistic (e.g., Laing et al., 2009) samples, only two studies have directly compared the relative contributions of RSMB and IS subdomains to both internalising and externalising behaviours. One, a longitudinal study of a community sample, reported that at age 6 years, RSMB, but not IS, predicted externalising behaviours, while IS but not RSMB predicted internalising behaviours (Carrington et al., 2024). The latter pattern was consistent with findings from a sample of children with Down's syndrome (Evans et al., 2014). Together, these scarce findings demonstrate the differential contribution of RRB subdomains to different aspects of emotional and behavioural difficulty and highlight the need for further research to explore their relative contribution to internalising and externalising behaviours.

Social communication difficulties incorporate a range of verbal and non‐verbal impairments. ‘Pragmatics’ refers to how language is used to communicate, including broader aspects of social interaction in social communication (Levinson, 1983). Pragmatic language difficulties can include difficulties in the use of context to interpret and use language, such as understanding non‐literal language, providing coherent but not over‐informative descriptions, conversational turn‐taking and topic management (Matthews et al., 2018). Previous studies using a parent questionnaire to measure children's communication (Bishop, 2003; Wellnitz et al., 2021) with autistic (e.g., Rodas et al., 2017) and community samples (e.g., Ketelaars et al., 2010) have shown strong associations between pragmatic language difficulties and internalising and/or externalising behaviours. Nevertheless, there is only limited evidence on whether pragmatic difficulties show a different pattern of association with internalising versus externalising behaviours, especially in non‐clinical samples (Dall et al., 2022).

In summary, a review of research in clinical and non‐clinical samples shows that while a very limited number of studies have examined differential associations between features of autism and internalising and externalising in non‐clinical samples by studying either RRBs or pragmatic language difficulties separately, these associations have not been examined simultaneously within the same sample. The current study will therefore characterise for the first time the differential and relative contribution of RRB subdomains and social communication (pragmatic language difficulties) to internalising and externalising behaviours in a non‐clinical sample. Importantly, the current research may also provide evidence to pinpoint whether features of different neurodevelopmental domains or subdomains associate differently with either internalising or externalising behaviours. Such differential associations would indicate more specific relationships between autistic features and emotional and behavioural difficulties than previously thought, a finding that might be applicable across different populations.

By setting aside diagnostic categories to examine these associations in a school‐identified sample recruited on the basis of functional needs, the study has relevance for research by enabling reduction of clinical selection biases of specific diagnoses. It also opens potential for translational relevance to education services by highlighting children likely to have vulnerabilities in behaviour and learning, where support needs are high, but where children may be less likely to receive support without a diagnosed condition. This research may therefore enable more targeted neurodevelopmental support for children with recognised emotional and behavioural difficulties, but no neurodevelopmental diagnoses.

As internalising and externalising behaviours are themselves known to be highly related to each other (Willner et al., 2016), our analytic strategy followed the recommendations of Achenbach et al. (2016) by (a) statistically exploring these two types of behaviour in separate hierarchical linear regression models, and (b) controlling for externalising behaviours whilst examining internalising behaviours and vice versa. Our investigation therefore first controlled for the covarying effect of internalising or externalising behaviours. Within this step, we also controlled for age, sex, and socio‐economic status (SES) to replicate evidence reported by Carrington et al. (2024) that associations between RRBs and internalising and externalising behaviours depended on the age at which RRBs were measured, as well as evidence of an association between sex and externalising behaviours (although RRBs remained the strongest predictor). Our next step was to examine the contribution of RRBs to these behaviours by testing previous evidence of differential contributions of RRB subdomains to different aspects of emotional and behavioural difficulties. Based on findings reported by Carrington et al. (2024), it was hypothesised that RSMB but not IS would predict externalising behaviours, while IS but not RSMB would predict internalising behaviours. Going beyond this, our final step was to examine the effects of pragmatic language on internalising or externalising behaviours over and above the contribution of RRBs. Given limited findings on the contributions of pragmatic language to both internalising and externalising behaviours, no specific hypotheses were made of a differential association for pragmatic language. Finally, given the paucity of evidence exploring the relative contributions of RRBs and pragmatic language, we ran a second set of analyses in which the order of entry of RRBs and pragmatic language was reversed. Entering pragmatic language in Step 2 allowed us to examine its contribution independently of RRBs, and entering RRBs in Step 3 revealed whether they made a significant contribution over and above pragmatic language. No specific hypotheses were made regarding whether RRBs or pragmatic language would make the strongest contribution to internalising or externalising behaviours.

## METHOD

### Participants

Children in the sample were recruited to the Neurodevelopment Assessment Unit (NDAU) at Cardiff University, Wales, following referrals from mainstream schools based on teachers' judgements of non‐specific emotional, behavioural or cognitive (e.g., memory, attention) difficulties. No child had a clinical diagnosis of neurodevelopmental and/or learning disorder. Only children who do not have a diagnosis of a DSM‐5 condition such as autism or ADHD can be referred to the NDAU research unit. No data are purposively collected on whether children will eventually be referred for clinical diagnosis. This is because the NDAU was designed to follow a dimensional framework (e.g., RDoC) and is not measuring clinical status (e.g., DSM‐5).

The data for the current sample was first reported by Keating, et al. (2024). There were 143 children in the original cross‐sectional study with complete data available for the RRB and pragmatic language measures. However, seven of these participants were missing data for the measure of internalising and externalising behaviours (six had no data while one further participant was missing data for nine [36%] items) and were consequently excluded. The final sample consisted of 136 children, aged 6 and 7 years, with a mean age of 6 years and 11 months (SD = 6.83 months), 80 (58.82%) of whom were male. Approximately 89% of parents identified as Welsh, English, Scottish, or Irish and 44.6% were classed as low SES, as defined by scores within the highest two quintiles of the Welsh Index of Multiple Deprivation (mean = 905.4, SD = 574.28; Welsh Government, 2019). Due to the teacher‐referral process and use of secondary data, it was not possible to involve people with lived experience in the early stages of the study. However, a parent with lived experience commented on the manuscript.

### Measures

*The Welsh Index of Multiple Deprivation* (Welsh Government, 2019) estimates relative deprivation within Wales using postal address. Areas are ranked from the most deprived (ranked 1) to the least deprived (ranked 1909). SES can be described on the basis of either rank order or quartiles.

*The Repetitive Behaviour Questionnaire‐2* (RBQ‐2; Leekam et al., 2007) is a 20‐item caregiver‐report questionnaire designed to assess the presence of restricted and repetitive patterns of behaviour over the past month. Each item is scored on a 3‐point scale, indicating the frequency or severity of the behaviour described (never/rarely, mild/occasional, marked/notable). The RBQ‐2 comprises of RSMB and IS subscales, which showed excellent internal consistency in the current sample (*α* = 0.86 and 0.88, respectively). Further details of the RBQ‐2 item numbers and subscales are available in Keating et al. (2023, 2024) and in Table S1.

*The Strengths and Difficulties Questionnaire* (SDQ; Goodman, 1997) is a 25‐item questionnaire, which measures emotional and behavioural difficulties in children seen over the last 6 months. Four of the five primary subscales can be combined to form two broad domains, internalising (emotional and peer problems) and externalising (conduct problems and hyperactivity), and higher total scores indicate greater difficulty (see Table S1 for item descriptions).

*The Child Communication Checklist‐2 revised* (CCC‐R; Wellnitz et al., 2021) is a reduced 39‐item revision of the original CCC‐2 (Bishop, 2003) that captures strengths and weakness in different aspects of communication. The CCC‐R summarises raw item scores for a pragmatic subscale (26 items; max score 78) derived from the original subscales of the CCC‐2 (D‐J), and a grammatical‐semantic subscale (13 items; max score 39). Higher scores indicate greater difficulty. The CCC‐R pragmatics scale include broader aspects of social communication, and due to the focus of the current study, only data from this scale was used.

### Procedure

Children and their families were seen at the NDAU at Cardiff University. Each child completed a battery of task‐based assessments, while their parent or guardian completed a series of questionnaires, including the RBQ‐2, CCC‐2, and SDQ (for further details, see Keating et al., 2024, 2023). The study was approved by the Cardiff University School of Psychology Research Ethics Committee; all parents/guardians provided informed consent before participating, while children gave their assent.

### Analysis plan

Statistical analyses were completed in IBM SPSS 28. Demographic and descriptive statistics were summarised for the RBQ‐2, SDQ, and CCC‐R. All variables failed to meet assumptions for normality according to the Shapiro–Wilk test; consequently, all analyses were bootstrapped with 1000 resamples to generate more reliable and robust statistics.

Non‐parametric correlations were first conducted between demographic variables and RBQ, SDQ, and CCC‐R subscales. A series of hierarchical linear regression analyses were then conducted to examine the contribution of RRBs and pragmatic language to externalising and internalising behaviours separately.

In the model predicting externalising behaviours, internalising behaviours were controlled for in Step 1 while in the model predicting internalising behaviours, externalising behaviours were controlled for in Step 1. Demographic variables were also included in Step 1. In the first set of analyses, Step 2 was designed to directly replicate the analyses conducted by Carrington et al. (2024). Both RRB subdomains were entered at this step to examine each subdomain (RSMB and IS as predictors) as well as the overall effect of RRBs (expressed as the proportion of variance explained and the *Fchange* statistic). Pragmatic language difficulty score was then entered in Step 3, the final step, in order to examine its contribution beyond RRBs. To further clarify the relative contribution of RRBs and social communication (pragmatic language) to internalising and externalising behaviours, a second set of analyses were conducted, in which pragmatic language was entered in Step 2 and RRBs were entered in the third step.

## RESULTS

Mean scores and standard deviations for each of the measures are presented in Table S1. There were no significant correlations with SES, and although higher RSMB and IS scores were associated with younger age (IS: *r*_s_ = −0.20, *p* < 0.05; RSMB: *r*_s_ = −0.20, *p* < 0.05) and male sex (IS: *r*_s_ = −0.18, *p* < 0.05; RSMB: *r*_s_ = −0.19, *p* < 0.05), these associations did not survive the correction for multiple comparisons (*p* > 0.003; Table S2). Both RRB subdomains were highly correlated with pragmatic language (IS: *r*_s_ = 0.61, *p* < 0.005; RSMB: *r*_s_ = 0.65, *p* < 0.005), and both RRBs and pragmatic language were moderately correlated with both internalising and externalising difficulties and with pragmatic language (*r*_s_ between 0.35 and 0.52, *p* < 0.005; Table S3).

Given that both age and sex were significantly (*p* < 0.05) correlated with RSMB and IS prior to correction for multiple comparisons, we conducted follow‐up bootstrapped partial correlations between the RRB subdomains and both internalising and externalising behaviours controlling for age and sex. The RRB‐externalising and RRB‐internalising associations remained significant for both RRB subdomains whether controlling for age alone, sex alone, or both age and sex together (correlation coefficients ranged from 0.35 to 0.54, all *p* < 0.001).

Regression models were run both with and without the inclusion of age and sex in Step 1 (SES was not included given the absence of any significant correlations). Given converging evidence of the limited impact of demographic variables on the variables of interest, demographic variables were not included within the main regression models. For comparison, regression analyses including age and sex are reported in Supporting Information S1: Appendix S1, Tables S4–S7; importantly, the overall pattern of results was unchanged at both the model and individual predictor level, with highly comparable effect sizes.

In the first set of regression analyses, we entered RRBs before pragmatic language to replicate analyses reported by Carrington et al. (2024) and then examined the added contribution of pragmatic language. Table 1 shows the results for externalising behaviours. In Step 1, internalising behaviours were entered as a control variable and accounted for 10% of the variance (*F*_(1, 134)_ = 16.20, *p* < 0.001). The inclusion of RRBs in Step 2 significantly improved the model fit (*F*change_(2, 132)_ = 8.69, *p* < 0.001), accounting for an additional 10.4% of the variance. At this stage, only RSMB was significant (*t* = 2.98, *p* < 0.001, *β* = 0.33). The inclusion of pragmatic language in Step 3 accounted for an additional 6.5% of the variance, and significantly improved the model (*F*change_(1, 131)_ = 11.84, *p* < 0.001). In this final step, only pragmatic language significantly predicted externalising behaviours (*t* = 3.44, *p* < 0.005, *β* = 0.35).

**TABLE 1:** Regression models predicting externalising behaviours, with RRBs entered before pragmatic language.

	Adjusted *R* ^2^	Δ*R* ^2^	*B*	SE B	*β*
**Step 1**	0.10**				
Internalising			0.35	0.09	0.33**
**Step 2**	0.19**	0.10**			
Internalising			0.17	0.10	0.16
RSMB			2.75	0.90	0.33**
IS			0.39	0.85	0.05
**Step 3**	0.26**	0.07**			
Internalising			0.13	0.10	0.12
RSMB			1.53	0.88	0.18
IS			−0.28	0.85	−0.04
Pragmatic language			0.09	0.03	0.35*

Abbreviations: IS, insistence on sameness; RSMB, repetitive sensory and motor behaviours; SE B, standard error in B.

*p < 0.05, **p < 0.001.

Table 2 shows the results for internalising behaviours. In Step 1, the entry of externalising behaviours as a control variable accounted for 10% of the variance (*F*_(1, 134)_ = 16.20, *p* < 0.001). The inclusion of RRBs in Step 2 significantly improved the model (*F*change_(1, 132)_ = 15.98, *p* < 0.001), and accounted for a further 17.4% of the variance. At this stage only IS significantly predicted internalising behaviours (*t* = 3.43, *p* < 0.005, *β* = 0.36). The inclusion of pragmatic language in Step 3 accounted for <1% of additional variance and did not improve the model. In this final step, IS remained the only significant predictor (*t* = 3.02, *p* < 0.005, *β* = 0.32).

**TABLE 2:** Regression models predicting internalising behaviours, with RRBs entered before pragmatic language.

	Adjusted *R* ^2^	Δ*R* ^2^	*B*	SE B	*β*
**Step 1**	0.10**				
Externalising			0.31	0.07	0.33*
**Step 2**	0.27**	0.17**			
Externalising			0.14	0.07	0.15
RSMB			1.01	0.82	0.13
IS			2.54	0.73	0.36*
**Step 3**	0.27**	0.01			
Externalising			0.11	0.07	0.12
RSMB			0.69	0.84	0.09
IS			2.31	0.75	0.32*
Pragmatic language			0.03	0.02	0.12

Abbreviations: IS, insistence on sameness; RSMB, repetitive sensory and motor behaviours; SE B, standard error in B.

*p < 0.05, **p < 0.001.

In the second set of regressions, pragmatic language was entered before RRBs. Table 3 shows the model examining externalising behaviours. In Step 1, the entry of internalising behaviours as a control accounted for 10.10% of the variance (*F*_(1, 134)_ = 16.20, *p* < 0.001). The inclusion of pragmatic language in Step 2 significantly improved the model (*F*change_(1, 133)_ = 27.65, *p* < 0.001), accounting for an additional 15.40% of the variance. Moreover, pragmatic language was the only significant predictor of externalising behaviours in this step (*t* = 5.26, *p* < 0.001, *β* = 0.43). Finally, the inclusion of RRBs in Step 3 did not significantly improve the model, accounting for just 1.6% of the variance. Moreover, in this step, only pragmatic language (*t* = 3.44, *p* < 0.005, *β* = 0.35) significantly predicted externalising behaviours.

**TABLE 3:** Regression models predicting externalising behaviours, with pragmatic language entered before RRBs.

	Adjusted *R* ^2^	Δ*R* ^2^	*B*	SE B	*β*
**Step 1**	0.10**				
Internalising			0.35	0.09	0.33**
**Step 2**	0.25**	0.15**			
Internalising			0.16	0.10	0.14
Pragmatic language			0.12	0.02	0.43**
**Step 3**	0.26	0.02			
Internalising			0.13	0.10	0.12
Pragmatic language			0.09	0.03	0.35*
RSMB			1.53	0.90	0.18
IS			−0.28	0.82	−0.04

Abbreviations: IS, insistence on sameness; RSMB, repetitive sensory and motor behaviours; SE B, standard error in B.

*p < 0.05, **p < 0.001.

Table 4 shows the model examining internalising behaviours. In Step 1, the entry of externalising behaviours as a control, accounted for 10% of the variance (*F*_(1, 134)_ = 16.20, *p* < 0.001). The inclusion of pragmatic language in Step 2 significantly improved the model (*F*change_(1, 133)_ = 15.27, *p* < 0.001), accounting for an additional 9.2% of the variance. In this step, only pragmatic language significantly predicted internalising behaviours (*t* = 3.91, *p* < 0.005, *β* = 0.35). Finally, the inclusion of RRBs in Step 3 further improved the model (*F*change_(2, 131)_ = 8.19, *p* < 0.001), accounting for an additional 9% of the variance. In this final step, pragmatic language was no longer significant and only IS significantly predicted internalising behaviours (*t* = 3.02, *p* < 0.001, *β* = .32).

**TABLE 4:** Regression models predicting internalising behaviours, with pragmatic language entered before RRBs.

	Adjusted *R* ^2^	Δ*R* ^2^	*B*	SE B	*β*
**Step 1**	0.10**				
Externalising			0.31	0.07	0.33**
**Step 2**	0.19**	0.09**			
Externalising			0.15	0.08	0.16
Pragmatic language			0.09	0.02	0.35*
**Step 3**	0.27**	0.09**			
Externalising			0.11	0.08	0.12
Pragmatic language			0.03	0.03	0.12
RSMB			0.69	0.83	0.09
IS			2.31	0.75	0.32**

Abbreviations: IS, insistence on sameness; RSMB, repetitive sensory and motor behaviours; SE B, standard error in B.

*p < 0.05, **p < 0.001.

Thus, regardless of the order of entry, we found that pragmatic language difficulties/social communication made the strongest contribution to externalising behaviours, whereas IS made the strongest contribution to internalising behaviours.

## DISCUSSION

The current study provided new insights on the nature of the association between specific neurodevelopmental features and internalising and externalising behaviours, when examined simultaneously within a single sample of children. Consistent with previous findings, we found that both RRB subdomains and pragmatic language (as a measure of social communication) correlated moderately with internalising and externalising behaviours (bivariate correlations ranging from 0.39 to 0.52; Table S3). The regression analyses, however, highlighted new and distinctive key findings. First in simultaneous models entering both RRBs and pragmatic language, we found that pragmatic language difficulties showed unique associations with externalising. In contrast RRBs—driven by IS—showed unique associations with internalising. Second, specific analysis of RRB subdomains showed how RSMB and IS subtypes differentially associate with internalising and externalising; IS but not RSMB made a significant contribution to internalising, whilst RSMB but not IS made a significant contribution to externalising. Importantly, the specific effect of IS on internalising still held even when pragmatic language was entered. However, the RSMB subdomain only made a significant contribution when entered into the regression model before pragmatic language.

The neurodevelopmental features examined in this study are described as autistic symptoms within traditional categorical diagnostic systems in psychiatry (e.g., DSM‐5; APA, 2013). Nevertheless, these features—RRBs and pragmatic language—are dimensional constructs that are also found together in non‐diagnosed children (Frazier et al., 2014; Keating et al., 2023). Similarly, internalising and externalising behaviours are defined as dimensions in transdiagnostic research frameworks (e.g., Kotov et al., 2017, 2022; Michelini et al., 2024). Thus, by adopting functional recruitment (Astle et al., 2022) to examine behaviours independently of diagnoses, this study followed the principles of recent dimensional research (e.g., Cuthbert & Insel, 2013; Kotov et al., 2017). Moreover, by mutually adjusting for internalising and externalising within regression analyses, it was possible to meaningfully examine the independent contribution of neurodevelopmental features to variance in these behaviours. The results therefore replicate and extend findings reported in a community sample (Carrington et al., 2024), demonstrating the same pattern of associations for RRBs across a broader spectrum of neurodevelopment and adding new insights into differential associations with pragmatic language.

Future research should aim to further clarify the developmental trajectory of neurodevelopmental features independently of categorical diagnostic criteria, as these in turn may have impact on internalising and externalising behaviours. For example, it is suggested that RRBs support neural and motor function in infancy, reducing as more cognitive strategies of self‐regulation develop with age (Thelen, 1981). Therefore, RRBs later in development might signal difficulty with or differences in cognitive regulatory function (Carrington et al., 2024; Evans et al., 1999; Leekam et al., 2011), which may in turn impact internalising and/or externalising behaviour, depending on the RRB subdomain in question. With respect to pragmatic difficulties, it has been suggested that challenges with turn‐taking or initiating conversations within linguistic and social development can have consequences for externalising behaviours, which are subsequently adopted as compensatory strategies to achieve goals that cannot be met by typical channels of communication (Fagan & Iglesias, 2000; Miranda et al., 2023). Internalising behaviours, such as anxiety and social withdrawal, may also be a consequence of these communication challenges (Boonen et al., 2015).

The results also have implications for clinical and educational practice. First, they have immediate relevance for highlighting that children whose main presenting challenges are emotional and behavioural can also have co‐occurring neurodevelopmental features that are under‐recognised (Astle et al., 2022). Second, by identifying distinct patterns of associations connecting neurodevelopmental features with emotional and behavioural difficulties, the results may help target potential support pathways. Moreover, although internalising and externalising behaviours commonly co‐occur (Willner et al., 2016), the identification of associations that are adjusted for this covariance may allow for support specifically tailored around someone's individual profile of emotional and behavioural difficulty. Such support could be beneficial for children with any form of difficulty but may be particularly important for children with emotional and behavioural difficulties who are currently unable to access support because they fall through the cracks created by the categorical diagnostic system. For example, if a child exhibits externalising behaviours, such as hyperactivity or conduct problems, it may be important to consider whether there are also pragmatic language difficulties. Although research findings to date lend support to evidence of an association between externalising behaviours and pragmatic language (e.g., Ketelaars et al., 2010), there has been only limited research examining the efficacy of pragmatic language interventions in decreasing externalising behaviour.

While support with pragmatic language development may also help children with internalising behaviours such as anxiety, the current results indicate a stronger role for IS RRBs. These results build on substantial evidence of an association between IS and anxiety (Baribeau et al., 2020; Lidstone et al., 2014; Rodgers et al., 2012; Spackman et al., 2023). One potential explanation for this association is that engaging in behaviours that maintain ‘sameness’ may serve to reduce uncertainty and thus anxiety. Intolerance of uncertainty (IU) is associated with anxiety in children and young people (Osmanagaoglu et al., 2018), and has also been found to mediate the association between IS and anxiety in autistic adults (Hwang et al., 2020). It seems plausible, therefore, that interventions focused on IU could be beneficial more broadly, and that persistent IS behaviours may signal the need to consider such interventions. Given that RRBs may serve a regulatory purpose for some children (e.g., Collis et al., 2022), interventions should not necessarily focus on reducing RRBs, but rather in supporting the difficulty (e.g., IU) they may signal. Importantly, the potential support pathways identified here are independent of specific categorical diagnoses. This study, therefore, provides evidence supporting recent calls to move towards a needs‐led rather than diagnosis‐led approach to neurodevelopmental conditions (e.g., Michelini et al., 2024).

### Limitations

Despite several strengths, this study also has some limitations. First, although the functional recruitment design (Astle et al., 2022) is a strength, the sample was from a relatively deprived background and, by design, included a narrow age‐range, both of which limit generalisability. While the contributions of age, sex, and SES were found to be minimal, future research should examine the effect of these variables in a more demographically heterogeneous sample. Moreover, although all children were referred for school‐identified emotional, behavioural, and/or cognitive difficulties, variation in the profile of difficulties leading to the referral was not considered within this study, as our aim was to adopt a dimensional approach as opposed to applying any form of categorisation. Nevertheless, mean scores for both internalising and externalising behaviours (Table S1) were higher than UK norms for 5–10‐year‐olds (https://www.sdqinfo.org/norms/UKSchoolNorm.html), indicating that it was unlikely any child presented with only cognitive concerns. Future research might also consider other potential confounds, such as whether any children had received any additional educational or behavioural support, as such support might influence the manifestation of both internalising and externalising behaviours, as well as the neurodevelopmental features examined in this study. Consideration of parental mental health may also be informative, given evidence that children's emotional and behavioural difficulties are associated with parental wellbeing and mental health (e.g., D’Onofrio et al., 2007; Eley et al., 2015; Ford et al., 2004; Goodman et al., 2011).

An important concern is the issue of shared methods variance, given the reliance on one source of data (parent/caregiver report) for each participant. Thus, it will be important to further replicate and extend findings reported here with multiple informants and longitudinal samples, utilising a multi‐modal and multi‐method assessment approach. However, it is important to note that distinct patterns of correlations appeared even when associated variables were controlled, suggesting that the results capture variance of interest, and not just spurious measurement effects. The study was also cross‐sectional in design; thus while we could examine predictions in a statistical sense (e.g., whether one variable predicted variance in another), it was not possible to examine whether RRBs or social communication might predict internalising or externalising behaviours later in development. Another limitation is that although we sampled a greater range of developmental neurodiversity than studies focused on specific clinical or community samples, a comprehensive examination of the transdiagnostic associations between neurodevelopmental features and internalising and externalising behaviours should also include neurodevelopmental features more commonly associated with other conditions (e.g., ADHD, Obsessive Compulsive Disorder (OCD), genetic conditions).

## CONCLUSIONS

Our transdiagnostic approach enabled characterisation of distinct associations between neurodevelopmental features of autism and internalising and externalising behaviours in a diverse, non‐clinical group. As proposed by others aiming to clarify the role of neurodevelopmental features within transdiagnostic frameworks (Michelini et al., 2024), this approach may help inform future research directions and identify target areas for the support of children with emotional and behavioural needs. Our results therefore have implications both for transdiagnostic research and for children's clinical and education support services.

## AUTHOR CONTRIBUTIONS

**Sarah J. Carrington**: Conceptualization; writing—original draft; methodology; formal analysis; writing—review and editing. **Jennifer Keating**: Conceptualization; data curation; writing—review and editing; methodology. **Mirko Uljarević**: Formal analysis; methodology; writing—review and editing. **Kirsten Abbot‐Smith**: Writing—review and editing; methodology. **Catherine R. G. Jones**: Funding acquisition; data curation; writing—review and editing. **Susan R. Leekam**: Conceptualization; funding acquisition; writing—review and editing; writing—original draft; methodology; data curation; formal analysis.

## CONFLICT OF INTEREST STATEMENT

The authors declare no conflicts of interest.

## ETHICAL CONSIDERATIONS

Written informed consent to participate in this study was provided by the participants' legal guardian/next of kin, while children gave informed assent. The study was performed in line with the principles of the Declaration of Helsinki. It was reviewed and approved by School of Psychology Ethics Committee (Date: 25/11/2021; Approval number: EC.16.10.11.4592GR).

## Supporting information

Supporting Information S1

## Data Availability

The data that support the findings of this study are available from both the corresponding authors upon reasonable request. The conditions of our ethics approval do not permit public archiving of anonymised study data. Readers seeking access to the data should also contact the NDAU team representative (Catherine Jones) or the local ethics committee at the School of Psychology, Cardiff University. Access will be granted to named individuals in accordance with ethical procedures governing the reuse of sensitive data. Specifically, requestors must complete a formal data sharing agreement with Catherine Jones.
