# Supplementary material for: Understanding the associations between neurodevelopmental features and internalising and externalising behaviours: A transdiagnostic approach
Source: JCPP Adv. 2026 Mar 8:e70110. Online ahead of print. doi: 10.1002/jcv2.70110 (PMC13338984; doi:10.1002/jcv2.70110)
Supplement: Supplementary file 1 — Supporting Information S1 [file JCV2-9999-e70110-s001.pdf]

Understanding the associations between neurodevelopmental features and internalising and externalising behaviours: a transdiagnostic approach

Supporting Information

Mean scores for each of the measures are presented in Table S1. Participants had significantly higher rates of IS than RSMB (Z=-3.67, p<.001), and significantly more externalising than internalising difficulties (Z=-8.24, p<.001).

Table S1. Descriptive statistics (n=136)

Measure (potential range)	Minimum	Maximum	Median	Inter-quartile range	Mean (SD)
**RBQ-2**					
RSMB (1-3)	1	3	1.47	.078	1.58 (.52)
IS (1-3)	1	3	1.63	.88	1.72 (.56)
**CCCR**					
Pragmatic language (0-78)	.00	68	27.5	25.5	27.29 (16.14)
**SDQ**					
Internalising (0-20)	0	17	6.00	5.75	6.92 (4.01)
Externalising (0-20)	2	20	12.00	5.75	11.74 (4.31)

Abbreviations: RBQ-2=Repetitive Behaviour Questionnaire-2; RSMB=repetitive sensory and motor behaviours; IS=insistence on sameness, CCC-R=Revised Child Communication Checklist-2; SDQ=Strengths and Difficulties Questionnaire (Wellnitz et al., 2021).

Note 1: RBQ-2: The RSMB subscale includes items 1-6 and 8-10. The IS subscale includes items 11 and 13-19. Items 7 and 12 and 20 are not included in subscales. Subscale scores range from 0-3 as they are calculated as a mean of constituent items . For details see Leekam et al., 2007.

Note 2: CCC-R: The Pragmatic Scale includes the following items from the CCC-2: 5, 7, 8, 11, 14, 15, 18, 19, 20, 22, 23, 25, 26, 28, 31, 33, 35, 37, 39, 40, 41, 42, 45, 48, 49, 50. For details see Wellnitz et al., (2021).

Note 3: SDQ: The internalising domain includes items 3, 6, 8, 11, 13, 14, 16, 19, 23, 24. The externalising domain includes items 2, 5, 7, 10, 12, 15, 18, 21, 22, 25. All items are scored on a three-point Likert scale from “0” (not true) to “2” (certainly true). Domain scores are calculated as a total score across all constituent items.

Table S2. Spearman's correlation analyses between demographic variables and RRB subtypes

	Age	SES#	Sex
**SES**	-.02		
**Sex**	.03	.15	
**RSMB**	-.20*	-.07	-.19*
**IS**	-.20*	-.10	-.18*
**Pragmatic language**	-.13	-.05	-.01
**Internalising**	.06	-.03	.00
**Externalising**	-.11	-.03	-.04

*p<.05; **p<.003

Note 2: A Bonferonni correction for multiple comparisons was conducted (.05/18=.003). #SES data were not available for 6 participants; consequently, all correlations involving SES were conducted with n=130, whilst the remainder involved the whole sample (n=136). Age was coded in months, SES and sex as binary male/female, SES was coded as a rank score from the Welsh Index of Multiple Deprivation (WIMD).

Table S3. Spearman's correlation analyses of RRB subtypes, pragmatic language, and internalising and externalising

	RSMB	IS	Pragmatic language	Internalising
**IS**	.70**			
**Pragmatic language**	.65**	.61**		
**Internalising**	.46**	.52**	.40**	
**Externalising**	.44**	.39**	.46**	.35**

*p<.05; **p<.005

Note 3: Non-parametric correlations examined the associations between repetitive behaviours (IS and RSMB), emotional and behavioural difficulties (internalising and externalising), and pragmatic language. A Bonferroni correction for multiple comparisons was conducted (.05/10 = .005).

Appendix S1. Results for regression analyses including age and sex in Step 1

In the first set of regression analyses, we entered RRBs before pragmatic language. Table S4 shows the results for externalising behaviours. In step 1, internalising behaviours, age, and sex were entered as control variables and accounted for 10.4% of the variance ($F_{(3, 132)}$= 6.20, p<.001). Only internalising significantly predicted externalising behaviours (t=4.14, p<.001, β=.34). The inclusion of RRBs in Step 2 significantly improved the model fit (Fchange$_{(2, 130)}$= 7.44, p<.001), accounting for an additional 9% of the variance. At this stage only RSMB was significant (t=2.92, p<.005, β=.33). The inclusion of pragmatic language in step 3 accounted for an additional 6.5% of the variance, and significantly improved the model (Fchange$_{(1, 129)}$= 11.59, p<.001). In this final step, only pragmatic language significantly predicted externalising behaviours (t=3.40, p<.005, β=.35).

Table S4. Regression models predicting externalising behaviours

	Adjusted R^2	ΔR^2	B	SEB	β
**Step 1**	.10**				
Internalising			.36	.09	.34**
Age			-.08	.05	-.12
Sex			-.33	.70	-.04
**Step 2**	.18**	.09**			
Internalising			.18	.10	.16
Age			-.02	.05	-.03
Sex			2.44	.70	.03
RSMB			2.74	.94	.33*
IS			.36	.81	.05
**Step 3**	.25**	.07**			
Internalising			.14	.10	.13
Age			-.02	.05	-.03
Sex			-.13	.69	-.02
RSMB			1.46	.97	.17
IS			-.36	.83	-.05
Pragmatic language			.09	.03	.35*

**p<.001; *p<.05. Abbreviations: RSMB=repetitive sensory and motor behaviours; IS=Insistence on Sameness

Table S5 shows the results for internalising behaviours. In Step 1, the entry of externalising behaviours, age, and sex as a control variable accounted for 10.1% of the variance ($F_{(3, 132)}$= 6.06, p<.001). Only externalising significantly predicted internalising behaviours (t=4.14, p<.001, β=.34). The inclusion of RRBs in step 2 significantly improved the model ($F\text{change}_{(2, 130)}$= 20.46, p<.001), and accounted for a further 21% of the variance. At this stage both age (t=2.82, p<.05, β=.21) and IS significantly predicted internalising behaviours (t=3.87, p<.001, β=.39). The inclusion of pragmatic language in the step 3 accounted for less than 1% of additional variance and did not improve the model. In this final step, both age (t=2.78, p<.05, β=.21) and IS (t=3.49, p<.001, β=.37) remained the only significant predictors

Table S5. Regression models predicting internalising behaviours

	**Adjusted R^2**	**ΔR^2**	**B**	**SEB**	**β**
**Step 1**	.10**				
Externalising			.32	.08	.34**
Age			.07	.05	.11
Sex			.10	.68	.15
**Step 2**	.31**	.21**			
Externalising			.13	.08	.14
Age			.12	.05	.21*
Sex			.8	.59	.10
RSMB			1.28	.83	.16
IS			2.81	.73	.39**
**Step 3**	.30**	.00			
Externalising			.11	.08	.12
Age			.12	.05	.21*
Sex			.71	.59	.09
RSMB			1.03	.88	.13
IS			2.63	.77	.37**
Pragmatic language			.02	.02	.09

**p<.001; *p<.05. Abbreviations: RSMB=repetitive sensory and motor behaviours; IS=Insistence on Sameness

In the second set of regressions, pragmatic language was entered before RRBs. Table S6 shows the model examining externalising behaviours. In Step 1, the entry of internalising behaviours, age, and sex as controls accounted for 10.4% of the variance ($F_{(3, 132)}$= 6.20, p<.001). Only internalising significantly predicted externalising behaviours (t=4.14, p<.005, β=.34). The inclusion of pragmatic language in Step 2 significantly improved the model ($Fchange_{(1, 131)}$= 25.34, p<.001), accounting for an additional 14.2% of the variance. Moreover, pragmatic language was the only significant predictor of externalising behaviours in this step (t=5.03, p<.001, β=.42). Finally, the inclusion of RRBs in Step 3 did not significantly improve the model, accounting for just 1.3% of the variance. Moreover, in this step, only pragmatic language (t=3.40, p<.005, β=.35) significantly predicted externalising behaviours.

Table S6. Regression models predicting externalising behaviours

	**Adjusted R^2**	**ΔR^2**	**B**	**SEB**	**β**
**Step 1**	.10**				
Internalising			.36	.09	.34**
Age			-.08	.05	-.12
Sex			-.33	.71	-.04
**Step 2**	.24**	.14**			
Internalising			.16	.10	.15
Age			-.03	.05	-.05
Sex			-.34	.66	-.04
Pragmatic			.12	.03	.42**
**Step 3**	.25	.01			
Internalising			.14	.10	.13
Age			-.02	.05	-.03
Sex			-.13	.72	-.02
Pragmatic			.09	.03	.35*
RSMB			1.46	.96	.17
IS			-.36	.87	-.05

**p<.001; *p<.05. Abbreviations: RSMB=repetitive sensory and motor behaviours; IS=Insistence on Sameness

Table S6 shows the model examining internalising behaviours. In Step 1, the entry of externalising behaviours as a control, accounted for 10.1% of the variance ($F_{(3, 132)}$= 6.06, p<.001). Only externalising significantly predicted internalising behaviours (t=4.14, p<.005, β=.34). The inclusion of pragmatic language in Step 2 significantly improved the model ($Fchange_{(1, 131)}$= 16.60, p<.001), accounting for an additional 9.9% of the variance. In this step, only pragmatic language significantly predicted internalising behaviours (t=4.08, p<.001, β=.36). Finally, the inclusion of RRBs in Step 3 further improved the model ($Fchange_{(2, 129)}$= 11.5, p<.001), accounting for an additional 11.5% of the variance. In this final step, pragmatic language was no longer significant and both age (t=2.78, p<.05, β=.21) and IS significantly predicted internalising behaviours (t=3.49, p<.005, β=.37).

Table S7. Regression models predicting internalising behaviours

	Adjusted R^2	ΔR^2	B	SEB	β
**Step 1**	.10**				
Externalising			.32	.08	.34*
Age			.07	.05	.11
Sex			.10	.65	.01
**Step 2**	.20**	.10**			
Externalising			.15	.08	.16
Age			.08	.05	.14
Sex			.04	.61	.01
Pragmatic			.09	.02	.36**
**Step 3**	.30**	.12**			
Externalising			.11	.08	.12
Age			.12	.05	.21*
Sex			.71	.58	.09
Pragmatic			.02	.03	.09
RSMB			1.03	.89	1.31
IS			2.63	.74	.37*

**p<.001; *p<.05. Abbreviations: RSMB=repetitive sensory and motor behaviours; IS=Insistence on Sameness
